# A New Licensed Quadrivalent Antileptospiral Canine Vaccine Prevents Mortality, Clinical Signs, Infection, Bacterial Excretion, Renal Carriage and Renal Lesions Caused by *Leptospira* Australis Experimental Challenge

**DOI:** 10.3390/vaccines12101104

**Published:** 2024-09-26

**Authors:** Jérôme Bouvet, Carine Segouffin Cariou, Frantz Oberli, Anne-Laure Guiot, Lionel Cupillard

**Affiliations:** 1 Boehringer Ingelheim, Centre de Recherche de Saint-Vulbas, Parc Industriel de la Plaine de l’Ain, 805 Allée des Cyprès, 01150 Saint-Vulbas, France; frantz.oberli@boehringer-ingelheim.com; 2 Boehringer Ingelheim, Lyon Porte des Alpes, 99 rue de l’Aviation, 69800 Saint-Priest, France; carine.cariou@boehringer-ingelheim.com (C.S.C.); lionel.cupillard@boehringer-ingelheim.com (L.C.); 3 CPB—Conseils en Pharmacie et Biologie, 2 Place des Quatre Vierges, 69110 Sainte Foy les Lyon, France; a-l.guiot@cegetel.net

**Keywords:** *Leptospira*, Australis, dog, vaccine, challenge

## Abstract

Background: *L.* Australis is one of the most prevalent *Leptospira* strains infecting dogs, leading, in natural conditions, to severe life-threatening cases. Objective: The objective was to evaluate the onset and duration of immunity (OOI and DOI) induced by a new licensed quadrivalent antileptospiral vaccine (EURICAN^®^ L4) including four *Leptospira* components (Canicola, Icterohaemorrhagiae, Grippotyphosa and Australis) against *L.* Australis. To this end, a severe *L.* Australis challenge model was developed, using a canine strain recently isolated from the field. Material and Methods: Seven- to ten-week-old puppies received two doses of the vaccine four weeks apart and were challenged with an *L.* Australis isolate two weeks (OOI) and 12 months (DOI) later. Mortality, clinical signs, leptospiremia, leptospiruria, renal carriage, and renal lesions were assessed after challenge. Results: The challenge induced multiple severe clinical signs in controls, leading to the death or euthanasia of 83% of puppies and 57% of adults. In controls, leptospiremia was detected in all dogs, leptospiruria in 67% of puppies and 86% of adults, kidneys tested positive for *Leptospira* in 83% of puppies and 71% of adults, and kidney lesions were observed in 100% of puppies and 86% of adults. In addition, thrombocytopenia associated with increased concentrations of urea, creatinine, and aspartate aminotransferase was recorded in controls displaying severe clinical signs. In both OOI and DOI studies, none of the vaccinates had clinical signs, no *Leptospira* was detected in blood, urine, and kidney samples, and no kidney lesions were observed in vaccinates. No significant changes in hematological and biochemical parameters in vaccinates were recorded. Conclusion: EURICAN^®^ L4 was shown to induce quick and long-lasting protection against a severe *L.* Australis infectious challenge, preventing mortality, clinical signs, infection, bacterial excretion, renal lesions, and renal carriage.

## 1. Introduction

Leptospirosis is one of the most widespread zoonotic diseases affecting most mammalian species, including humans. It is caused by a spirochete of the genus *Leptospira*, including more than 250 pathogenic serovars [1]. Animals or humans become infected by direct contact of abraded skin or mucosal surfaces with urine of the carrier mammals or indirect contact with contaminated soil and water [2,3]. In dogs, a wide range of clinical manifestations, from subclinical to fatal illness, have been reported [2,4,5]. Male adult dogs with street access that come into contact with environmental water have a higher risk of exposure to *Leptospira* [6]. Several serovars can infect dogs, with a variable geographical distribution depending in particular on the maintenance host and vaccination. Historically, Canicola and Icterohaemorrhagiae were the first main serovars associated with canine leptospirosis. However, changes in the epidemiology of canine leptospirosis have occurred and new serovars such as Grippotyphosa and Australis in Europe, and Grippotyphosa and Pomona in North America have emerged [7]. Several sero-epidemiological studies conducted in a number of European countries, such as France [8,9], Italy [10], Switzerland [11,12], Greece [13], Spain [14], Germany [15], and Sweden [16], documented the frequent exposure of dogs to the serogroup Australis, mainly serovar Bratislava, with or without clinical signs. In a recent study, 239 blood and urine samples from dogs suspected of leptospirosis and detected positive for lipL32 by PCR were analyzed. It was shown that the most prevalent serogroups in Europe were Icterohaemorrhagiae (53%) and Australis (13%) [17]. Exposure to *L.* Australis was also reported in the United States [18], Japan [19,20,21], Brazil [22], and Australia [23]. Maintenance hosts for the serovar Bratislava are pigs, horses, hedgehogs, and probably dogs [3,7].

Bivalent vaccines including serovars Canicola and Icterohaemorrhagiae have been available since the 1960s for the protection of dogs. Because immunity to *Leptospira* is strongly restricted to the homologous serovar or closely related serovars [1,2], the emergence of two main epidemiologically relevant serovars led to the inclusion of Grippotyphosa and Australis along with the historical ones, Canicola and Icterohaemorrhagiae, in leptospirosis vaccines in the 2010s [24,25,26,27,28].

In March 2023, a new quadrivalent antileptospiral vaccine (EURICAN^®^ L4) was granted marketing authorization in Europe.

In this paper, we describe the level of protection of this new vaccine for the *L.* Australis component both in onset and duration of immunity, using a severe challenge model with a canine strain recently isolated in France from a dog suffering from acute leptospirosis [29]. Clinical signs, leptospiremia, leptospiruria, renal carriage, and renal lesions were assessed after *L.* Australis challenge performed 2 weeks and 12 months after primary vaccination.

## 2. Material and Methods

Two different studies were conducted: an onset of immunity study (OOI study) and a duration of immunity study (DOI study).

### 2.1. Vaccines and Solvent

EURICAN^®^ DAPPi (Boehringer Ingelheim Vetmedica GmbH, Ingelheim am Rhein, Germany) is a multi-component non-adjuvanted freeze-dried vaccine containing 4 attenuated live viruses (canine distemper virus, canine adenovirus type 2, canine parvovirus type 2, and canine parainfluenza virus).

EURICAN^®^ L4 (Boehringer Ingelheim Vetmedica GmbH) is a liquid non-adjuvanted vaccine composed of inactivated cultures of 4 *Leptospira* serovars (Canicola, Icterohaemorrhagiae, Grippotyphosa, and Bratislava). The quadrivalent antileptospiral vaccine used in the studies was formulated at the minimum protective dose for each *Leptospira* component.

EURICAN^®^ solvent (Boehringer Ingelheim Vetmedica GmbH).

### 2.2. Animals

A total of 26 conventional Beagle dogs, provided by a commercial supplier, were included in the studies, i.e., 6 males and 6 females in the OOI study, and 4 males and 10 females in the DOI study. They were aged between 7 and 10 weeks on day 0 (D0) and tested negative for agglutinating antibodies (microscopic agglutination titer < 1/50) against the main serovars of pathogenic *Leptospira*, i.e., Icterohaemorrhagiae, Canicola, Grippotyphosa, Sejroe, Hardjo, Hebdomadis, Pomona, Australis, and Autumnalis. Dogs were randomly assigned according to their litter, birth date, and sex to one group of dogs vaccinated against leptospirosis (named vaccinates) and one group of controls in each study, with 6 dogs per group in the OOI study and 7 dogs per group in the DOI study.

Vaccinates and controls were housed together during the vaccination phase and after challenge.

In each study, vaccinates and controls were not housed by group. The 2 groups of each study were mixed in the housing boxes.

### 2.3. Experimental Design

In both OOI and DOI studies, the schedule of vaccination against leptospirosis was similar, with one subcutaneous injection of the EURICAN^®^ DAPPi-L4 vaccine on D0 (between the shoulder blades) and on D28 (left shoulder), 4 weeks apart. Control dogs received EURICAN^®^ DAPPi (reconstituted with EURICAN^®^ solvent) on D0 and D28.

All dogs were challenged on D45 (T0) in the OOI study and on D398 (T0) in the DOI study.

**Figure 1:**
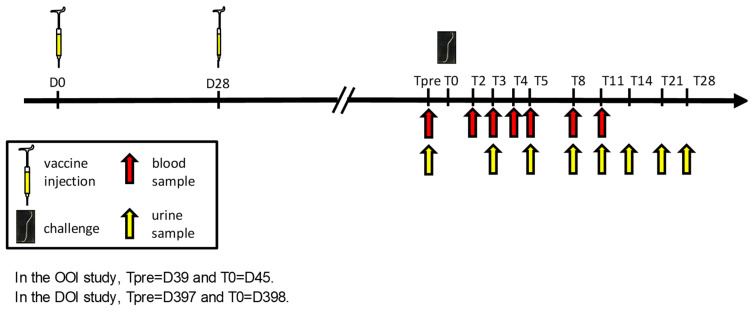
Study design.

The study design and schedule of sample collection are summarized in Figure 1. The study design complied with Monograph 0447, “Canine leptospirosis vaccine (inactivated)”, of the European Pharmacopoeia.

Studies were carried out in accordance with the EU Directive 2010/63/EU for animal experiments and the National Research Council’s Guide for the Care and Use of Laboratory Animals. The Ethics Committee of Boehringer-Ingelheim Animal Health France, CE013, agreed by the Research Ministry on 3 January 2023, has approved the clinical studies which have been conducted in compliance with the project authorization 2019071817016518_V2 on 10 September 2019, for 5 years. Endpoints were defined to avoid unnecessary suffering of diseased dogs.

The staff performing clinical monitoring and laboratory analyses was blinded to the treatment group.

### 2.4. Challenge

The challenge strain was isolated from a dog with severe clinical signs of acute leptospirosis (acute renal failure, anorexia, dehydration, abdominal pain) through a collaborative study between Boehringer Ingelheim and the National Veterinary School of Lyon. The serogroup Australis and serovar Bratislava were confirmed by the National Reference Center for *Leptospira* (CNRL Pasteur Institute, Paris, France) using molecular analysis.

After an initial culture in Ellinghausen–McCullough–Johnson–Harris (EMJH) medium, the challenge strain was passaged twice in hamsters. After checking the bacterial vitality and bacterial titer of the challenge suspension, each dog received 1.2 (DOI study) or 1.4 (OOI study) × 10^9^ leptospires by the intra-peritoneal route.

### 2.5. Clinical Monitoring

All dogs were observed daily for 28 days after challenge for signs consistent with leptospirosis. Six types of signs (general condition, dehydration, ocular signs, vomiting, diarrhea, and cutaneo-mucosal signs) were evaluated daily. Clinical signs were scored (see Table 1). Rectal temperatures were recorded daily for 7 days after challenge. Dogs were weighed once a week. Once the first clinical signs appeared, clinical examination was performed twice a day. Any dogs displaying serious and irreversible clinical signs that could lead to suffering were humanely euthanized, based on predefined endpoints (hypothermia, shock, general condition score 2, vomiting or diarrhea score 2 at three successive clinical examinations, daily score ≥ 9).

### 2.6. Sample Collection and Laboratory Analyses

For serology, blood was collected at the time of first vaccination (D0), before challenge (D39 for the OOI study, and D397 for the DOI study), and 28 days after challenge (or at the time of death/euthanasia). Antibodies specific to the serogroup Australis were measured by a blocking ELISA, as described by Cariou et al. [30]. The same monoclonal antibody was used as a capture and conjugate antibody. It was produced at Boehringer Ingelheim Animal Health and was shown to be specific to the lipopolysaccharide (LPS) of the serogroup Australis. Antibody titers were calculated at 50% of the optical density (OD_50_) and expressed as log_10_ OD_50_. The threshold of positivity was set at 0.30 log_10_ OD_50_.

For hematology (platelet count, in EDTA tubes) and blood chemistry (urea, creatinine, aspartate aminotransferase [AST], in dry tubes), blood was regularly collected before and after challenge (see Figure 1).

For the detection of *Leptospira*, blood (in heparin tubes) and urine were regularly collected before and after challenge (see Figure 1). Urine was obtained after subcutaneous injection of furosemide (2.5–5 mg/kg bodyweight) [31] via urinary catheterization, cystocentesis or direct bladder tap at the time of necropsy. Blood and urine samples were stored at 5 °C until testing. Kidney samples were aseptically collected at the time of necropsy. Approximately 2 cm^3^ of the organ tissue was placed in 5 mL of PBS without Ca^2+^ and Mg^2+^, then frozen at −20 °C until testing. The detection of *Leptospira* in blood, urine, and kidney samples was carried out using a quantitative real-time PCR assay targeting the lipL32 gene, as described by Blanchard et al. [32]. Each sample was tested in duplicate, and the result was expressed as negative (no detection of lipL32 in both assay samples) or positive (at least one assay sample with detection of lipL32).

After euthanasia or death, dogs were necropsied. Samples of kidneys were fixed with 10% buffered formalin, then processed for microscopic examination following standard procedures (Hemalun–Eosine–Safran staining).

### 2.7. Analyses of the Results

#### 2.7.1. Clinical Score

A Global Clinical Score (GCS) was derived from the assessments of each Individual Clinical Sign (ICS) measured on each dog throughout the monitoring period (number of days of monitoring = ndm) by taking into account the effective length of the monitoring period in the case of death/euthanasia of the dog (effective number of days of monitoring = ndme).

For each dog (i), GCS was defined as the sum of the ICS (k) over the day of measurement (j) and was computed as follows:

$${\mathrm{GCS}}_{i}=\sum_{j}\sum_{k}ICS_{j,k}\times \mathrm{ndm}/{\mathrm{ndme}}_{i}$$

#### 2.7.2. Hematological and Biochemical Data

Hematological and biochemical data were analyzed qualitatively.

Thrombocyte count < 150 × 10^9^/L, creatinine concentration > 135 µmol/L, and urea concentration > 7 mmol/L were considered abnormal values.

For AST, due to baseline values being higher than normal values observed in several vaccinated and control dogs before challenge, the analysis was based on the increase from baseline.

#### 2.7.3. Statistical Analyses

All analyses were conducted using SAS software (SAS Institute, Cary, NC, USA; version 9.4). The level of significance was set at 0.05.

Vaccinated and control groups were compared using a Barnard exact test for the frequency of dead/euthanized dogs, frequency of dogs with clinical signs, leptospiremia, leptospiruria, *Leptospira* detected in kidneys, and kidney lesions.

## 3. Results

### 3.1. Serology

No ELISA antibodies against *L.* Australis were detected in any dogs before the first vaccine injection, and control dogs remained seronegative until challenge in both studies.

In the OOI study, all vaccinates had seroconverted before challenge. In the DOI study, no antibodies were detected before challenge.

After challenge, *L.* Australis antibodies were detected in all controls and vaccinates, except in two vaccinates in the OOI study (Table 2).

### 3.2. Clinical Signs

Before challenge, all dogs remained healthy and gained weight after vaccination in both studies.

**Figure 2:**
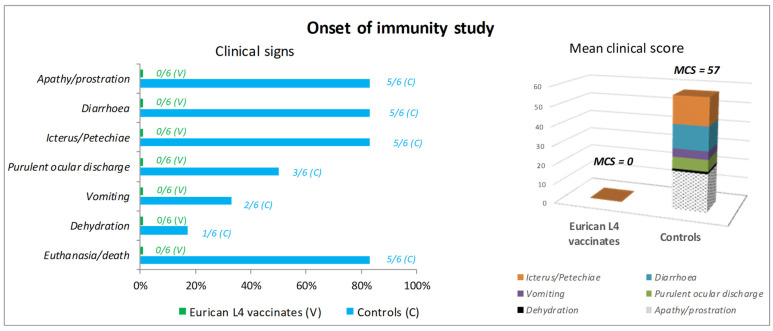
Onset of immunity. Clinical signs and clinical score after challenge.

In the OOI study, five out of six controls experienced, from day 4 post-challenge, a severe impairment of their general condition (prostration) associated with digestive signs (bloody diarrhea in five puppies and severe vomiting in two puppies), cutaneo-mucosal signs (marked icterus in five puppies) and ocular signs (purulent discharge in three puppies). Among these five controls, four puppies were humanely euthanized 4 (two dogs) or 5 (two dogs) days after challenge, and one puppy was found dead 5 days after challenge. The last control dog did not show any clinical signs. No hyperthermia was recorded in controls. The mean clinical score was 57 in controls (Figure 2). In contrast, neither clinical signs nor hyperthermia were observed in the six vaccinates, which gradually gained weight after challenge.

**Figure 3:**
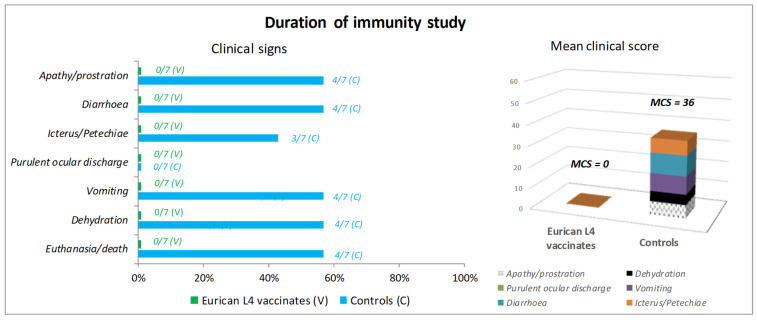
Duration of immunity. Clinical signs and clinical score after challenge.

In the DOI study, four controls developed, from day 4 post-challenge, severe clinical signs including impaired general condition (apathy/prostration), dehydration, digestive signs (diarrhea in four dogs which was bloody in three of them, and severe vomiting in four dogs), and marked icterus (three dogs). In addition, hyperthermia was recorded in two of them 3 days after challenge. Two other controls experienced mild clinical signs (slight dehydration or non-bloody diarrhea) once or twice. The last control dog did not show any clinical signs. The four controls with severe clinical signs were humanely euthanized 4 (two dogs), 6 (one dog) and 9 (one dog) days after challenge. The mean clinical score was 36 in controls. Four vaccinates remained in good general condition without any clinical signs after challenge. One vaccinate showed slight dehydration for 2 days. Slight dehydration and loose stools on the thermometer were recorded once in two other vaccinates. Weight remained stable in the seven vaccinates and three surviving controls at the end of the monitoring period. Since slight dehydration and/or loose stools, observed for 1 or 2 days in two controls and three vaccinates, were isolated (no other clinical signs), they were not considered as specific to leptospirosis and were not taken into account in the calculation of the clinical score for both groups (Figure 3).

In both studies, the prevention of mortality was demonstrated in the vaccinated group (OOI study: *p* = 0.003; DOI study, *p* = 0.012), as well as the prevention of clinical signs (OOI study, *p* = 0.003; DOI study, *p* = 0.012).

### 3.3. Hematology and Biochemistry

In the OOI study, the thrombocyte count of the five controls with severe clinical signs dropped after challenge, with values ranging from 10 to 29 × 10^9^/L on the day of death/euthanasia. Creatinine and urea concentrations of these five controls increased, with values ranging from 338 to 427 µmol/L for creatinine and from 33.5 to 55.7 mmol/L for urea on the day of death or euthanasia. These five controls also showed a marked increase from baseline of AST concentration (19- to 51-fold increase) after challenge. No changes in thrombocyte count, urea, creatinine, and AST values were observed in the sixth control dog.

In the DOI study, thrombocytopenia was observed in two controls, with the lowest values in the four dogs with severe clinical signs. Abnormal creatinine and urea concentrations were recorded for the four controls with severe clinical signs, reaching values from 371 to 1298 µmol/L for creatinine and 24.4 to 94.3 mmol/L for urea. AST values of these four controls were increased by 2.6- to 67-fold on the day of euthanasia, as compared to baseline. No changes in thrombocyte count, urea, creatinine, and AST values were observed in the seventh control dog.

Thrombocyte count, creatinine, urea, and AST concentrations remained stable in vaccinates in both studies, except for one vaccinate in the OOI study and two vaccinates in the DOI study with one or two urea values slightly above the normal concentration, and for two vaccinates in the DOI study with a single thrombocyte count slightly below the normal value. As microaggregates were observed in these blood samples and thrombocyte concentrations were twice as high the next day, these abnormal values were considered biologically not significant.

### 3.4. Detection of Leptospires in Blood, Urine, and Kidney

In the OOI study, leptospires were detected in the blood of all controls 2 days after challenge, then in five controls until their death/euthanasia. Four controls had positive urine and kidney samples. The fifth control dog with severe clinical signs became anuric, and no urine could be obtained on the 5th day, but its kidneys were detected positive for *Leptospira* by PCR at the time of its euthanasia (Table 3).

In the DOI study, all controls had at least two positive blood samples. Leptospiruria was recorded in six controls, and the kidneys of five controls were positive for *Leptospira*.

In contrast, no leptospires were detected in the blood, urine, and kidneys from vaccinates in either study (Table 3). The prevention of leptospiremia, leptospiruria, and renal carriage was demonstrated in the vaccinated group in both studies (*p* < 0.001 for leptospiremia and leptospiruria, and *p* < 0.004 for renal carriage).

### 3.5. Histopathological Findings

**Figure 4:**
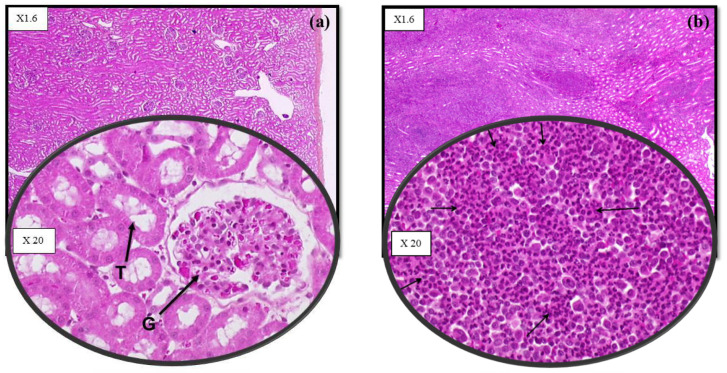
Histopathologic images from kidney samples. (**a**) Normal kidney of a vaccinated dog. Glomeruli (G) and tubules (T) have a normal appearance. (**b**) Kidney of a control dog experiencing severe clinical signs. Severe lesions of extensive interstitial nephritis with macrophage and neutrophile infiltrates (pyogranulomatous lesion) were observed (indicated by arrows).

In the OOI study, lesions were observed in both kidneys of the five controls with severe clinical signs. Kidney lesions were severe and consisted of diffuse cortical acute hemorrhages, multifocal interstitial polynuclear cell infiltration, and diffuse cortical tubular and glomerular necrosis. One kidney of the sixth control dog which survived the challenge showed mild lesions (multifocal interstitial cortical mononuclear cell infiltration) (Figure 4).

In the DOI study, six controls had mild or severe kidney lesions. Kidney lesions were similar to those observed in the OOI study. No kidney lesions were observed in the last control dog with no urine and kidney positive samples.

No kidney lesions were observed in any vaccinates in either study (Figure 4).

Prevention of kidney lesions was demonstrated in the vaccinated group for both studies (*p* < 0.001—see Table 4).

## 4. Discussion

This paper describes the level of protection of the *L.* Australis component of a new quadrivalent antileptospiral vaccine EURICAN^®^ L4 for both onset and duration of immunity, using a severe challenge model with a canine strain recently isolated in France.

To allow the assessment of the clinical protection provided by the vaccine, we developed an *L.* Australis challenge model inducing severe clinical signs, as conducted previously for *L.* Canicola, *L.* Icterohaemorrhagiae, and *L.* Grippotyphosa challenge models [28,33]. To this end, we selected an *L.* Australis strain isolated in France from a dog with acute leptospirosis. This challenge strain is different from the vaccinal strain isolated from a different region of France. Both strains belong to the serovar Bratislava. However, they have a different core genome sequence typing (cgST) determined at Pasteur Institute, which demonstrates that the strains are different from a genetic point of view and are from different lineages [29].

Our challenge model induced typical clinical signs of acute leptospirosis, such as marked icterus, bloody diarrhea, and vomiting, and a serious illness leading to the euthanasia or death of five out of six control puppies in the OOI study. It should be underlined that, although less frequent, severe multiple clinical signs were also observed in adult controls in the DOI study, requiring the euthanasia of four out of seven controls after challenge. This level of severity has not been obtained in previous *L.* Australis challenge models in either puppies or adult dogs [24,25,26,27], except in a recent report where one control puppy out of eight developed severe clinical signs while three other dogs had conjunctival suffusion only [34]. Besides the use of a recent field strain, several factors may contribute to the severity of our model. The dose of *Leptospira* inoculated in each dog (1.2 to 1.4 × 10^9^/dog) was at least 6 or 13 times higher than the dose used in the Klaasen study [34] (2 × 10^8^/dog) and Wilson studies [24,25] (9 × 10^7^/dog). Moreover, our challenge strain underwent a few passages in vitro after its isolation and was passaged twice in hamsters just before challenge. Our challenge model is representative of acute severe leptospirosis due to *L.* Australis in the field: severe life-threatening cases of leptospirosis due to *L.* Australis were reported in Italy [35,36], France [29], China [37], and Japan with a mortality rate as high as 83.3% [19,20,21].

In this challenge model, vaccinates were fully protected against severe clinical signs and death as soon as 2 weeks after the second dose of primary vaccination, and for at least 12 months. Since exposure to *L.* Australis is frequently reported in Europe and can induce a serious illness in the field, clinical protection is crucial. EURICAN^®^ L4 is the only vaccine which demonstrated significant prevention of severe clinical signs after an *L.* Australis challenge leading to the death or euthanasia of 83% of control puppies and 57% of adults. In the recent Klaasen study [34], no significant prevention of clinical signs was shown in the OOI study for the two other tetravalent *Leptospira* vaccines licensed in Europe, while clinical protection was demonstrated in a less severe challenge model for one of these vaccines [24,25], but not for the second [26,27].

For the detection of *Leptospira* in the blood, urine, and kidneys, we used a real-time PCR method targeting the gene of lipL32, an outer-membrane, surface-exposed lipoprotein, found exclusively in pathogenic leptospires. This PCR assay was shown to be rapid and specific for the detection of pathogenic *Leptospira* in urine and blood samples during vaccine efficacy studies, without a loss of sensitivity as compared to the culture method [32]. Leptospires were detected in the blood of all controls in both studies, while no vaccinates became positive. After infection, leptospires are disseminated to other organs via the hematogenous route [5]. Prevention of bacteriemia is therefore expected to reduce bacterial load in organs, in particular in the kidneys, contributing to reduced lesions and shedding.

Besides protecting against clinical signs and leptospiremia, vaccination should also prevent renal colonization and bacterial excretion in urine. Urine and/or kidneys were positive for *Leptospira* in 83% (OOI study) and 86% (DOI study) of controls. In contrast, no positive urine and kidney samples were detected in vaccinates in either study. Consistent with the PCR results from urine and kidney samples, renal lesions were observed in kidneys from 100% (OOI study) and 86% (DOI study) of controls, but not from vaccinates (0%). Lesions were the most severe in controls which were euthanized or died before the end of the study. These findings are comparable to renal lesions described during the acute phase of leptospirosis, such as acute interstitial nephritis with tubular and glomerular degeneration, and the infiltration of mononuclear cells, lymphocytes, plasma cells, and macrophages [3]. Prevention of renal carriage and bacterial excretion reduces environmental contamination and possible transmission to other animals and humans, and is thus important not only from an animal but also from a public health perspective [5]. Urinary shedding is not uncommon in asymptomatic dogs and was documented in 1.5% of dogs in upper Bavaria [38], 4% in Thailand [39], 7% in Ireland [40], 8% in the US [41], and in up to 20% in an endemic area in Brazil [42]. In a limited study conducted in France, 13.3% of asymptomatic dogs vaccinated with a bivalent vaccine had positive urine samples by PCR [43].

Hematological and biochemical analyses evidenced a drop in thrombocyte count, as well as increased concentrations of urea, creatinine, and AST in controls displaying severe clinical signs. Mild-to-severe thrombocytopenia is common in dogs with leptospirosis [5]. The biochemical abnormalities observed in our studies were also frequently described after natural exposure and experimental challenge [5,18,26,28,35], and support a kidney and liver impairment. No changes in these parameters were observed in vaccinates in either study, except for a few values slightly above or below normal values, which were observed occasionally. Their biological significance may be questioned because they were not associated with any clinical signs, leptospiremia, or leptospiruria.

Immunity induced by bacterin vaccines is generally based on antibodies directed mainly against the *Leptospira* LPS, a T-independent antigen, involving IgM antibodies with a lack of memory response [44]. Several studies have documented that vaccine-associated *Leptospira* microagglutination titers, as well as ELISA antibody titers, are generally low and short-lived, with no direct correlation between antibody titers and protection [25,28,30,33,45,46,47]. The studies described in this paper display a similar pattern with either short-lived or undetectable antibodies, confirming this absence of correlation. Recently, a study reported the induction of another type of immune response after annual revaccination against leptospirosis. Increased secretion of C-X-C motif chemokine ligand 10 (CXCL-10) and interferon gamma (IFN-γ), enhanced proliferation of CD4+ and CD8+ T cells, and expansion of a central memory CD4+ T-cell subset were shown in vaccinated dogs, suggesting the induction of a T-cell-mediated immune response, potentially contributing to protection [48].

## 5. Conclusions

*L.* Australis is one of the most prevalent *Leptospira* strains infecting dogs, leading, in natural conditions, to severe life-threatening cases.

EURICAN^®^L4 was shown to induce quick and long-lasting protection against a severe *L.* Australis infectious challenge, preventing mortality, clinical signs, infection, bacterial excretion, renal lesions, and renal carriage.

## Figures and Tables

**Table 1:** Scoring of clinical signs.

**GENERAL CONDITION**
0 = good—*animal in good health which plays and is attentive*
1 = apathy—*animal with lack of energy (when compared to its normal behavior) or animal which stays lying but responds to stimuli*
2 = prostration—*animal which stays lying without responding to stimuli* 10 = animal found dead
**DEHYDRATION**
0 = absence of dehydration
1 = slight dehydration
2 = severe dehydration
**OCULAR SIGNS**
0 = absence of purulent discharge
2 = purulent discharge
**DIGESTIVE SIGNS: VOMITING**
0 = absence of signs
1 = vomiting without abdominal pain
2 = severe vomiting alone or abdominal pain alone or severe vomiting with blood and/or abdominal pain
**DIGESTIVE SIGNS: DIARRHEA**
0 = absence of signs
1 = non-bloody diarrhea
2 = bloody diarrhea
**CUTANEO-MUCOSAL SIGNS**
0 = absence of signs
1 = slight icterus visible only on ocular mucosa
2 = obvious icterus (of all non-pigmented body surfaces) alone or hemorrhagic disorders (petechiae, suffusions, hemorrhages) alone or obvious icterus with mucosa congestion and/or hemorrhagic disorders

**Table 2:** ELISA antibody titers against *L.* Australis (in log_10_ OD_50_): mean and range (in brackets).

Study		Titer at Time of 1st Vaccination	Titer at Time of Challenge	Titer after Challenge *
Onset of immunity (OOI)	Vaccinates	neg	0.51 (0.38–0.74)	<0.64 (neg–0.86)
	Controls	neg	neg	1.58 (0.60–2.27)
Duration of immunity (DOI)	Vaccinates	neg	neg	1.52 (1.06–1.98)
	Controls	neg	neg	>1.78 (1.04–>2.41)
	neg: <0.30			

* Sampling on the day of euthanasia/death for some controls (between day 4 and day 9 after challenge) or at the end of the study (day 28 after challenge).

**Table 3:** Detection of *Leptospira* by PCR in blood, urine, and kidney in vaccinates (V) and controls (C) in the onset and duration of immunity studies.

		Days after Challenge													
		Blood						Urine							Kidney
Study	Group	2	3	4	5	8	11/12	3	5	8	11/12	14	21	28	Death/ Euthanasia
Onset of immunity (OOI)	V	0/6	0/6	0/6	0/6	0/6	0/6	0/6	0/6	0/6	0/6	0/6	0/6	0/6	0/6
	C	6/6	5/6	5/6	3/4	0/1	0/1	1/6	4/5	0/1	0/1	0/1	0/1	0/1	5/6
Duration of immunity (DOI)	V	0/7	0/7	0/7	0/7	0/7	0/7	0/7	0/7	0/7	0/7	0/7	0/7	0/7	0/7
	C	6/7	7/7	3/7	0/5	0/5	0/4	2/7	3/5	3/4	3/4	2/3	2/3	2/3	5/7

**Table 4:** Summary of main results (proportion of affected dogs after *L.* Australis challenge). Vaccine claims.

	Onset of Immunity			Duration of Immunity			
Parameter	V	C	*p* Value *	V	C	*p* Value *	Claim
Death	0%	83%	**0.003**	0%	57%	**0.012**	Prevents mortality
Clinical signs	0%	83%	**0.003**	0%	57%	**0.012**	Prevents clinical signs
Leptospiremia	0%	100%	**<0.001**	0%	100%	**<0.001**	Prevents infection
Leptospiruria	0%	67%	**0.011**	0%	86%	**<0.001**	Prevents bacterial excretion
Positive kidney	0%	83%	**0.003**	0%	71%	**0.004**	Prevents renal carriage
Kidney lesions	0%	100%	**<0.001**	0%	86%	**<0.001**	Prevents renal lesions

**V:** vaccinated dogs; **C:** control dogs. * in bold, significant difference between groups.

## Data Availability

Data sharing is not applicable to this article due to privacy reasons (data have been shared with European Medicines Agency).

## Abbreviations

OOI, onset of immunity; DOI, duration of immunity; L, leptospira; D, day; T, time; ELISA, enzyme-linked immunosorbent assay; PBS, phosphate-buffered saline; EMJH, Ellinghausen–McCullough–Johnson–Harris; LPS, lipopolysaccharide; AST, aspartate aminotransferase; GCS, Global Clinical Score; ICS, Individual Clinical Score; OD50, optical density 50%; CXCL-10, C-X-C motif chemokine ligand 10; IFN-γ, interferon gamma
